# An analytical approach to determine the optimal duration of continuous glucose monitoring data required to reliably estimate time in hypoglycemia

**DOI:** 10.1038/s41598-020-75079-5

**Published:** 2020-10-23

**Authors:** Nunzio Camerlingo, Martina Vettoretti, Andrea Facchinetti, Giovanni Sparacino, Julia K. Mader, Pratik Choudhary, Simone Del Favero

**Affiliations:** 1 Department of Information Engineering, University of Padova, 35131 Padova, Italy; 2 Division of Endocrinology and Diabetology, Medical University of Graz, 8036 Graz, Austria; 3 Department of Diabetes, School of Life Course Sciences, King’s College London, London, SE59RJ UK

**Keywords:** Applied mathematics, Biomedical engineering, Diabetes

## Abstract

Diabetes is a chronic metabolic disease that causes blood glucose (BG) concentration to make dangerous excursions outside its physiological range. Measuring the fraction of time spent by BG outside this range, and, specifically, the time-below-range (TBR), is a clinically common way to quantify the effectiveness of therapies. TBR is estimated from data recorded by continuous glucose monitoring (CGM) sensors, but the duration of CGM recording guaranteeing a reliable indicator is under debate in the literature. Here we framed the problem as random variable estimation problem and studied the convergence of the estimator, deriving a formula that links the TBR estimation error variance with the CGM recording length. Validation is performed on CGM data of 148 subjects with type-1-diabetes. First, we show the ability of the formula to predict the uncertainty of the TBR estimate in a single patient, using patient-specific parameters; then, we prove its applicability on population data, without the need of parameters individualization. The approach can be straightforwardly extended to other similar metrics, such as time-in-range and time-above-range, widely adopted by clinicians. This strengthens its potential utility in diabetes research, e.g., in the design of those clinical trials where minimal CGM monitoring duration is crucial in cost-effectiveness terms.

## Introduction

Diabetes is a chronic metabolic disease, affecting around 450 million people worldwide^1^, caused either by the autoimmune destruction of insulin secreting cell (Type 1 diabetes), or by malfunction in insulin secretion or action (Type 2 diabetes). Consequently, people with diabetes are subjected to undesirable excursions of blood glucose (BG) concentration outside the normal range, both in hyperglycemia ($BG>180$ mg/dL) and hypoglycemia ($BG<70$ mg/dL). In order to take proper countermeasures, such as exogenous insulin injections to contrast hyperglycemia, or fast-acting carbohydrates ingestion to balance hypoglycemia, individuals with diabetes need to frequently monitor BG by a portable device.

The most modern approach to BG monitoring relies on Continuous Glucose Monitoring (CGM) sensors, which are noninvasive, or minimally-invasive, devices able to produce glucose level readings almost continuously (e.g., every 5 min) for several consecutive days/weeks^2,3^. According to *T1D Exchange*, a research organization dedicated to improving care of people with Type 1 diabetes, data from 2016 to 2018 shows that 30% of individuals with Type 1 diabetes use a CGM^4^, and the number of users is expected to increase, especially when cheaper sensors will be available on the market (currently, in the US, the cheapest device costs approximately US$40 per week)^5^. In numerous recent studies^6–11^, CGM was seen clinically more effective than the traditional sparse monitoring made by fingerprick instruments. In research, CGM is now considered the state of the art system to gather data for evaluating the quality of diabetes therapies in clinical trials^12–14^. In particular, the recent consensus panel of Battelino et al.^14^ identified several CGM-based metrics which can be used to assess the efficacy of clinical interventions. Among them, given the fact that (the fear of) hypoglycemia is considered the major barrier in preventing diabetes complications^15,16^, the fraction of CGM readings below target glucose range (TBR):

1 $$\begin{aligned} TBR = \frac{1}{N} \sum _{k=1}^{N} CGM_{hypo}(k), \end{aligned}$$

where $CGM_{hypo}(k)=1$ if $CGM(k) < 70$ mg/dL, and it is 0 otherwise, is particularly important.

The number of data (*N*) considered in Eq. (1), related to the number of monitoring days, is critical. Scientists, clinicians and diabetes practitioners are well aware that evaluating TBR based on data collected in a too short monitoring period, i.e., *N* too small, might drive to conclusions flawed by physiological fluctuations (including illness and menstrual cycle) and lifestyle variability (travels, vacations, etc.). On the other hand, unnecessarily large values of *N* would result in an increase of experimental costs not justified by real benefits. This poses two questions which are the object of the present paper. The first question is: how precise is an estimate of TBR based on CGM data collected during a given temporal interval? The second one is: for how long should a patient be monitored to obtain a sufficiently reliable estimate of TBR?

Several published studies approached these two questions by simulating a retrospective correlation analysis^17–19^. For example, in Xing et al.^17^, the correlation coefficient between TBR computed over the data previously collected in a 3-month trial and the same metric computed over several shorter windows of increasing duration is calculated. Then, the minimal duration granting a correlation coefficient greater than a fixed threshold is selected. Based on the results obtained therein, the consensus of Battelino et al.^14^ recommended that 14 days of CGM monitoring are sufficient to obtain a clinically reliable estimate of TBR.

However, evaluations of this kind are only empiric and an analytical approach to the problem would be desirable. More important, it is easy to verify that the empirical method proposed in Ref.^17^ yields different results based on the duration of the reference dataset^20^.

In the present work, we present an analytical approach to determine the minimum duration that CGM recordings must have in order to produce TBR values that represent reliable estimates of exposure to hypoglycemia. The approach is based on studying the variance of the estimation error of TBR, resulting in a formula which allows determining the uncertainty of the TBR estimate. The formula can be extended in a straightforward fashion to other conceptually similar metrics, such as time-in-range and time-above-range, also widely adopted in diabetes research.

The paper is organized as follows: first we introduce the problem formulation and show the main result (Theorem 1), then we validate our findings on outpatient data. After presenting a second result (Theorem 2), some conclusions are drawn, with examples of possible applications. Finally we explain the methods that led to the results. Further information on methods and dataset can be found in the Supplementary Information.

## Results

### Problem formulation and analytical result

Glucose concentration can be modelled as a continuous random variable, that assumes values in the range $[0,+\infty ]$ mg/dL. CGM measurements are then modelled as a random process $g_k$, where $g_1, g_2, \ldots , g_k, \ldots , g_N$ are non-independent realizations of the process collected at time $t = k T_s$, $k = 1,\ldots , N$, where $T_s$ is the CGM sampling period. We are interested in estimating $p_h = {\mathbb {P}}[g_k < 70 \text {mg/dL}]$.

Let us introduce the following random process made of binary random variables:

2 $$\begin{aligned} h_k = {\mathbb {I}}_h(g_k) = {\left\{ \begin{array}{ll} 1, &{} \text{ if } g_k < \text{70 } \text {mg/dL}\\ 0, &{} \text{ otherwise } \end{array}\right. } \end{aligned}$$

that models samples in hypoglycemia, where ${\mathbb {I}}_h$ is the indicator function of hypoglycemia. Since $g_k$, $g_\ell$ are not independent, also $h_k$, $h_\ell$ are not independent.

By construction, $h_k \approx \text {B}(p_h)$ is a Bernoulli random variable of parameter $p_h$, that denote the probability of hypoglycemia. Mean and variance of $h_k$ are:

3 $$\begin{aligned} \begin{aligned} \mu&= {\mathbb {E}}[h_k] = p_h, \quad \forall k \\ \sigma ^2&= \text {var}[h_k] = p_h (1-p_h), \quad \forall k. \end{aligned} \end{aligned}$$

Let us assume an autoregressive structure of order 1 for $h_k$ (this hypothesis will be validated in this work). This means that two samples $k, \ell$, with $k \le \ell$, have the following autocovariance function:

4 $$\begin{aligned} \text {cov}[h_k,h_\ell ] = \alpha ^{\ell -k} \sigma ^2. \end{aligned}$$

In this framework, the TBR usually computed in clinical practice and defined in Eq. (1), can be seen as an estimator of $p_h$:

$$\begin{aligned} t(n) = \frac{1}{n} \sum _{k=1}^{n} h_k. \end{aligned}$$

It is easy to see that the estimator *t*(*n*) has the following proprieties:

1. Unbiased: $${\mathbb {E}}[t(n)]-p_h=0, \forall n.$$
2. Asymptotically consistent: $t(n) {\mathop {=}\limits ^{n \rightarrow \infty }} p_h$ (from the low of large numbers).

Finally, let us define the estimation error as:

$$\begin{aligned} \begin{aligned} e(n)&= t(n)-p_h \\&= \frac{1}{n} \sum _{k=1}^{n} (h_k-p_h). \end{aligned} \end{aligned}$$

In this context, the problem of determining the minimum duration of CGM that represents, with a certain accuracy, the time in hypoglycemia of an individual, translates in studying the convergence speed of $t(n) \rightarrow p_h$ and, therefore, how $\text {var}(e(n))$ decreases as *n* increases.

#### Theorem 1

Let $g_1, g_2, \ldots , g_N$ be a sequence of *N* non-independent CGM samples, obtained from the process $g_k$.

Let $h_k = {\mathbb {I}}(g_k)$ be the Bernoulli process describing samples in hypoglycemia, obtained as dichotomization of the process $g_k$.

Let $t(n) = \frac{1}{n} \sum _{k=1}^{n} h_k$ be the unbiased and asymptotically consistent estimator of the time in hypoglycemia $p_h$ and let $e(n) = t(n)-p_h$ be the estimation error.

Assume that $h_k$ can be modelled as an AR(1) process:

$$\begin{aligned} \text {cov}[h_k,h_\ell ] = \alpha ^{\ell -k} p_h(1-p_h). \end{aligned}$$

Then, the standard deviation of the estimation error is

5 $$\begin{aligned} \text {sd}[e(n)] = \sqrt{\frac{p_h(1-p_h)}{n} \bigg ( 1+\frac{2 \alpha }{1-\alpha } + \frac{2 \alpha }{n} \frac{\alpha ^n-1}{(1-\alpha )^2} \bigg )}. \end{aligned}$$

The proof is reported in the “Methods”.

*Notation* In this section and in the rest of the paper, we represent standard deviation and covariance of a random variable/random process with lower letters (sd and cov) while we will use capitalized letters for sample standard deviation and covariance (SD and COV), obtained averaging variable/process realization.

### Validation of the formula on real data

Validation will be performed first on a single-subject CGM trace, with $\alpha$ and $p_h$ specifically estimated for the patient under study, in order to validate the hypothesis of a AR(1). Nevertheless, patient specific values of $\alpha$ and $p_h$ are hardly known in practice. So, as second step, we will validate the formula using the population values $\alpha ^*$ and $p_h^*$ and test its ability to predict the error on several different patients.

Furthermore, when dealing with real data, the evaluation of the estimation error is complicated by the fact that the true subject time in hypoglycemia $p_h$ is unknown. Therefore, for each subject, in the estimation error evaluation, $p_h$ is approximated as ${\hat{p}}_h$ the time in hypoglycemia computed over the entire CGM trial,

$$\begin{aligned} {\hat{p}}_h = t(N), \qquad e(n)=t(n)-{{\hat{p}}}_h. \end{aligned}$$

However, as better discussed later on in this paper, this approximation is acceptable only when working with short estimation windows (i.e., $n<< N$), and thus in this section we limit the analysis to $n=1,\ldots ,n_{\max }$, with $n_{\max }<<N$ corresponding to 30 days.

#### Dataset

The analysis on CGM data is performed using a portion of data collected in the REPLACE-BG study^6^: a randomized trial which compares two different diabetes management approaches. The selected portion of the dataset involves 148 subjects with T1D for at least 1 year, monitored up to 6 months with a Dexcom G4 Platinum CGM sensor (Dexcom, Inc). Further study details and subjects extraction criteria are reported in the Supplementary Information.

#### Validation on CGM data of a single subject

In this section, we report the results obtained for a representative subject, specifically subject $\#16$. Similar results hold for any other subject.

**Figure 1:**
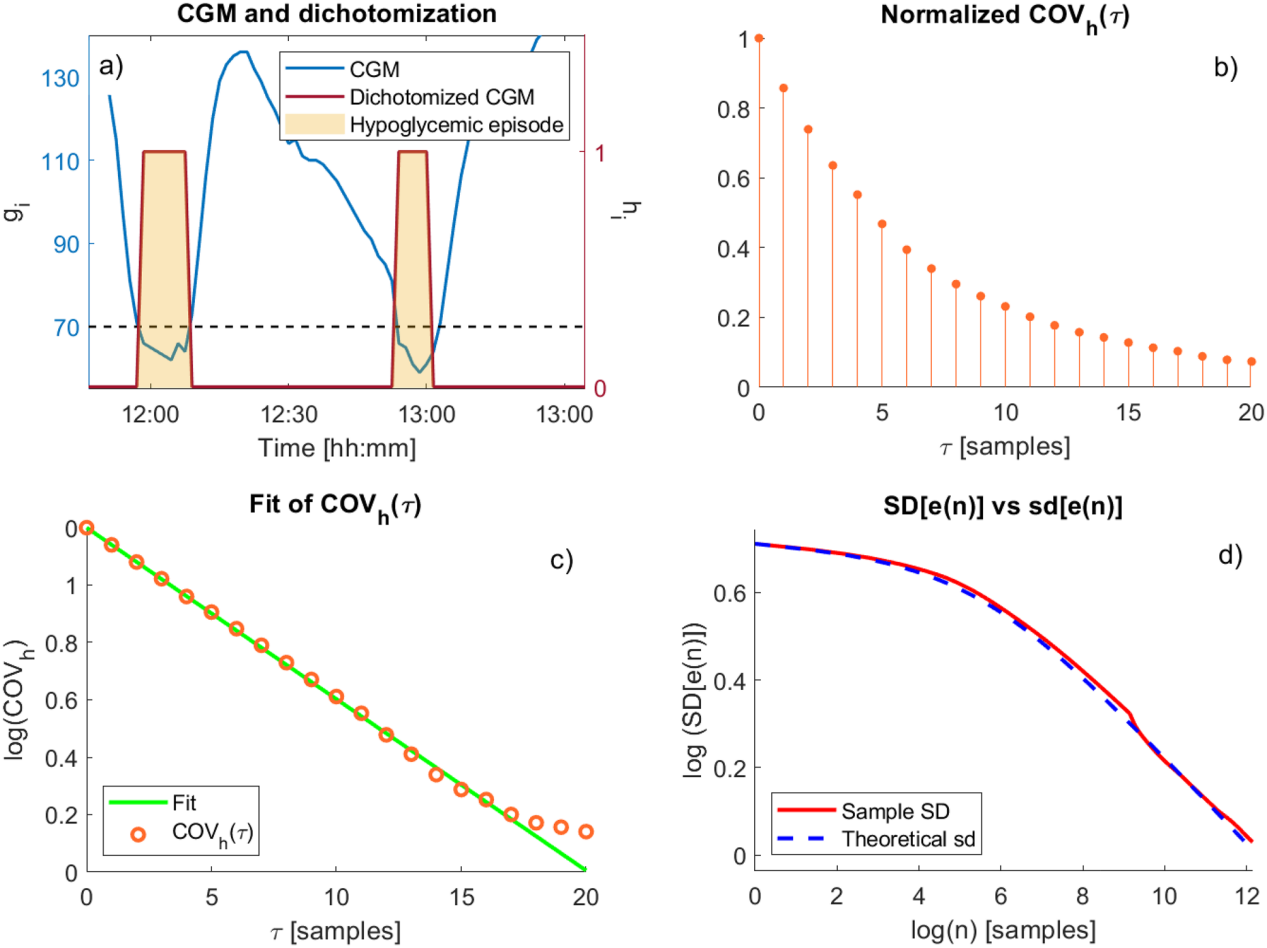
Single subject CGM data. (**a**) CGM data $g_i$, referred to left axis (blue), and dichotomized CGM data $h_i$, referred to right axis (orange). $h_i=1$ during the hypoglycemic events (yellow regions); (**b**) sample autocovariance function (COV($\tau$)) of the dichotomized CGM, normalized with respect to the sample variance of the process $\sigma ^2$. $\tau$, expressed in samples, represents the delay between two samples of the process; (**c**) exponential fit $\alpha ^\tau$ of the sample COV, reported in log scale; (**d**) sample standard deviation of the estimation error (red) and standard deviation obtained through the proposed formula (dashed blue), reported in log-log scale.

Initially, we dichotomize the CGM trace $g_k$ to obtain $h_k$, as in Eq. (2). A short portion of $g_k$ and $h_k$ is shown in Panel a of Fig. 1.

The objective of this section is to verify Eq. (4), i.e., checking that $h_k$ can be modelled as an AR(1). To do so, we compute the sample autocovariance function of the process, $COV_h(\tau )$, where $\tau$ is the lag between two samples:

$$\begin{aligned} COV_h(\tau ) = \frac{1}{N-\tau -1} \sum _{k=1}^{N-\tau }\left[ \left( h_k-\frac{1}{N}\sum _{\ell =1}^{N}h_\ell \right) \left( h_{k+\tau }-\frac{1}{N}\sum _{\ell =1}^{N}h_\ell \right) \right] \end{aligned}$$

that represents an estimate of the autocovariance function $\text {cov}_h(\tau )$ of the process, $\text {cov}_h(\tau ) = {\mathbb {E}}[(h_k-{\mathbb {E}}(h_k))(h_{k+\tau }-{\mathbb {E}}(h_{k+\tau }))]$.

The sample autocovariance function is reported in Panel b of Fig. 1, where the desired exponential shape is well visible. Although the hypothesis of AR(1) structure might be unsuitable to describe a CGM trace, which is often modelled as AR of a higher order^21,22^, such a structure seems to be appropriate to describe its dichotomized version.

We then fit a single exponential model $\alpha ^{\tau }$ to the autocovariance function, normalized with respect to the variance of the process, and estimate the parameter $\alpha$ using a Weighted Non Linear Least Squared (WNLLS) approach. For the example subject, we obtain $\alpha = 0.86$, and a good fit, as shown in panel c of Fig. 1, where data (orange dots) and model (green) are reported in logarithmic scale.

We also compute $\hat{p_h} = t(N)$, equal to 0.047, for the example subject, and consequently ${\hat{\sigma }}^2 = \hat{p_h} (1-\hat{p_h}) = 0.045$.

Finally, once estimated $\alpha$ and ${\hat{\sigma }}^2$, we are able to apply the theoretical formula to compute the standard deviation of the estimation error. Panel d of Fig. 1 shows in a log-log scale the prediction of the formula as a dashed blue line.

In the same panel, this theoretical prediction is compared with the sample standard deviation of the estimation error *e*(*n*; *i*) computed on all possible estimation windows of length $n=1,\ldots ,n_{\max }=30<<N$ in the patient under study

$$\begin{aligned} e(n;i)&=t(n;i)-{{\hat{p}}}_h= t(n;i)-t(N;i)\\ \text {SD}[e(n)]&=\sqrt{\frac{1}{M-1}\sum _{i=1}^{M}e(n;i)^2} \end{aligned}$$

with $i=1,\ldots ,M$ and *M* being the number of possible window. For example, for $n=1$ we have $M=N$ possible windows for each patient while for a generic *n* we have only $M=N-n+1$ possible windows of length *n*. Panel d of Fig. 1 shows that the proposed formula fits well the sample standard deviation estimation error *e*(*n*), proving that the assumption of an AR(1) structure is acceptable for $h_k$.

#### Validation on population CGM data

This section aims at verifying if our formula can be applied to an entire population.

**Figure 2:**
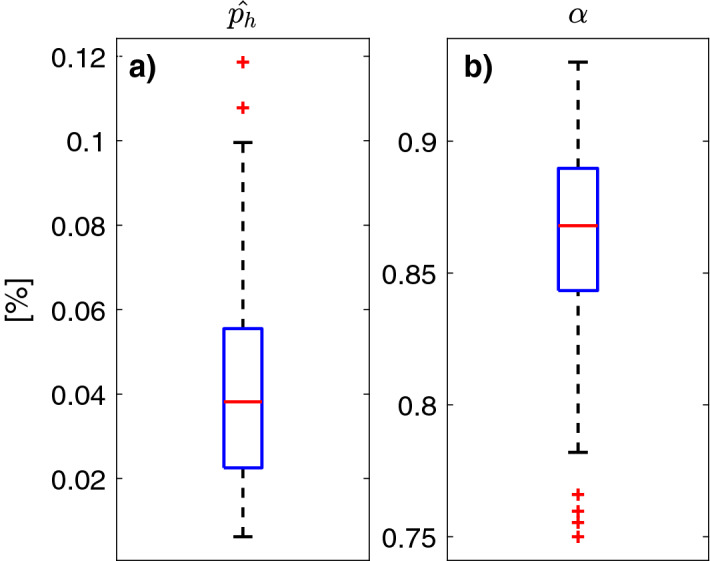
Boxplot representation of the distribution of the estimated ${{\hat{p}}}_h$ (**a**) and $\alpha$ (**b**), over the entire population. Red horizontal line represents median, the blue box marks the interquartile range, dashed black lines are the whiskers and red stars indicate outliers.

The previous analysis identified and employed a pair $({{\hat{p}}}_h,\alpha )$ for each subject. The distributions of these parameters in the population are shown in Fig. 2, ${{\hat{p}}}_h = 0.038\ [0.011 - 0.090]$ (median [$5{\text {th}}$ - $95{\text {th}}$ percentiles]) and $\alpha = 0.867\ [0.783 - 0.917]$.

We now consider the use of a single pair ($p_h^*$, $\alpha ^*$) in Eq. (5) to represent the entire population. We select the population $p_h^*$ as the distribution mean, equal to 0.043, while the population $\alpha ^*$ is selected as the $95{\text {th}}$ percentile of the distribution, equal to 0.917. This choice is due to the fact that the most correlated CGM traces (i.e., those with a higher $\alpha$) exhibit the slowest estimation error decrease, thus strongly influencing the estimation error in the population for large *n*.

Thus, we can use these population parameters to compute $\text {sd}[e(n)]$ using Eq. (5) that becomes

$$\begin{aligned} \text {sd}[e(n)] = 0.203\sqrt{\frac{23.1}{n}+266.2\frac{0.917^n-1}{n^2}}. \end{aligned}$$

**Figure 3:**
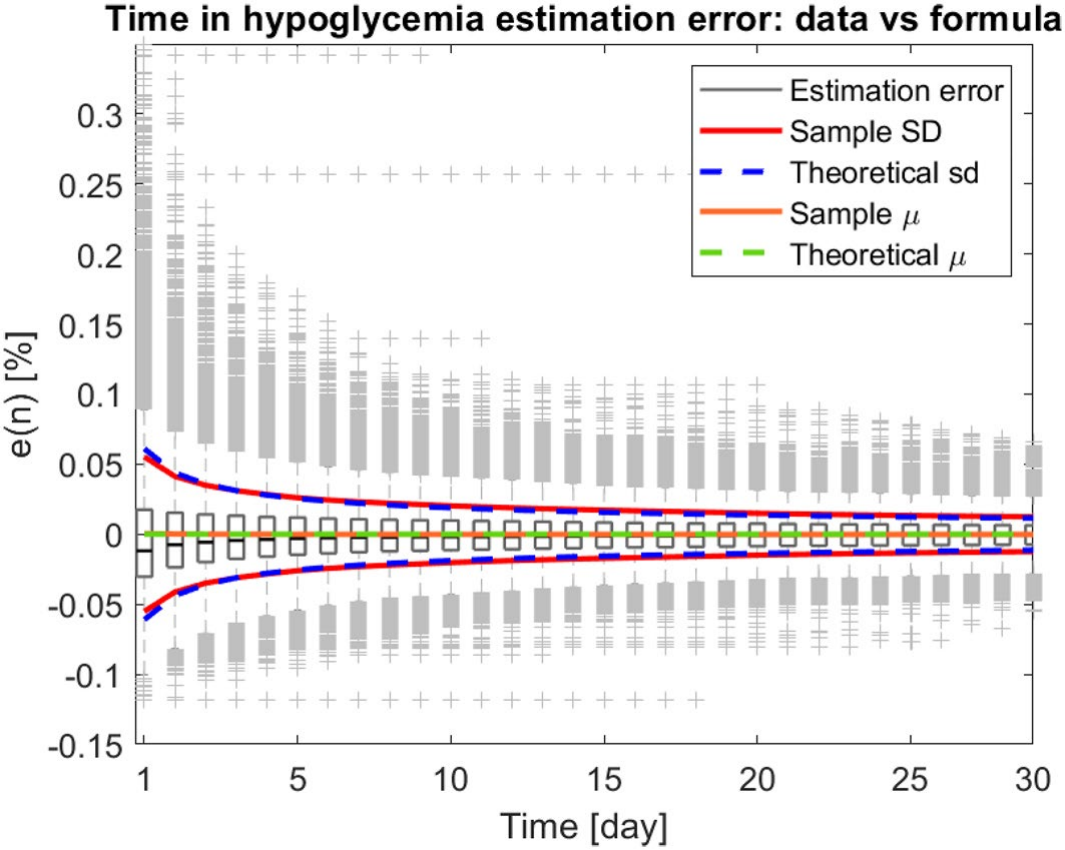
Time in hypoglycemia estimation error, for different window durations, for all the population under analysis. Mean and standard deviation of the estimation error are computed both on CGM data (orange for the mean, red for the standard deviation) and using the proposed formula of Eq. (5) (dashed green for the mean, dashed blue for the standard deviation).

Plotting the results of the above formula for various values of *n*, we obtain the dashed-blue line depicted in Fig. 3.

In the same figure, this theoretical prediction is compared with the sample standard deviation of the estimation error *e*(*n*; *i*) computed on all possible windows in all possible patients

$$\begin{aligned} e(n;i)&=t(n;i)-{{\hat{p}}}_h= t(n;i)-t(N;i)\\ \text {SD}[e(n)]&=\sqrt{\frac{1}{M-1}\sum _{i=1}^{M}e(n;i)^2}, \end{aligned}$$

where $i=1,\ldots ,M$ and *M* is the total number of possible windows. For example, for $n=1$ we have N possible windows for each patient, thus $M=N\cdot N_{\text {pat}}$, while for a generic *n* we have only $N-n+1$ possible windows of length *n* in each patient, so $M=(N-n+1)\cdot N_{\text {pat}}$. $\text {SD}[e(n)]$ is depicted in Fig. 3 as a red solid curve.

Moreover, in the figure we report the boxplots of *e*(*n*; *i*), $i=1,\dots ,M$ and depict also the sample mean of the estimation error (orange line) and its theoretical prediction, i.e., ${\mathbb {E}}[e(n)]=0$ as per Eq. (3) (green-dashed line). First of all, notice that sample mean and its theoretical prediction match well. More importantly, also the sample standard deviation and its prediction overlap very well. Therefore we can conclude that the proposed formula with the given population parameters is effectively able to describe the standard deviation of the estimation error for the overall population.

### Computing the sample standard deviation over long estimation windows

The formula of Theorem 1, assumes that true time in hypoglycemia $p_h$ of a subject is known. When analyzing real data, $p_h$ has to be approximated by ${{\hat{p}}}_h = t(N)$, the time in hypoglycemia computed over the entire CGM trial. By doing so, in real data validation we are computing $e(n;N)=t(n)-t(N)$ instead of $e(n)=t(n)-p_h$. In this section we will derive a formula for *sd*[*e*(*n*; *N*)].

**Figure 4:**
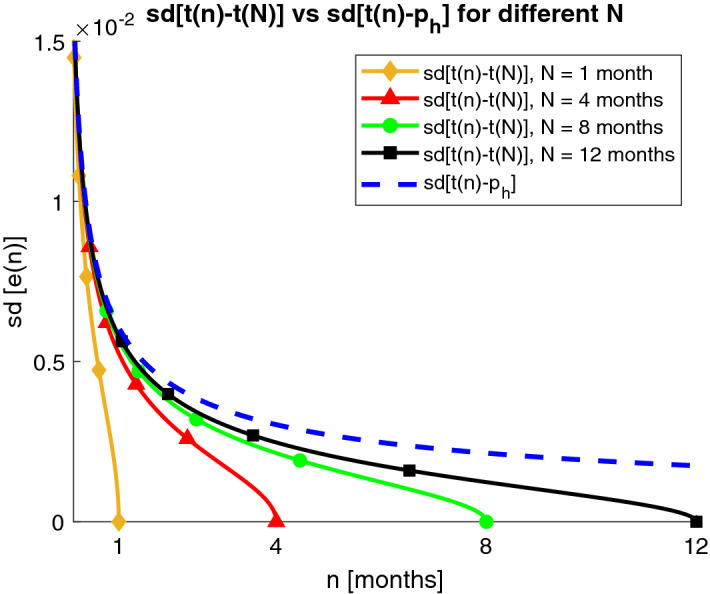
Standard deviation of the estimation error *sd*[*e*(*n*)] (dashed blue) and its approximation *sd*[*e*(*n*, *N*)] affected by the approximation ${{\hat{p}}}_h=t(N)$. The curves are obtained for different trial durations: $N=1$ month (diamond yellow), $N=4$ months (triangle red), $N=8$ months (circle green), $N=12$ months (square black). Moreover, $\alpha =\alpha *=0.917$ and $p_h=p_h*=0.043$ are used.

Then, we will compare *sd*[*e*(*n*; *N*)] with the true estimation error *sd*[*e*(*n*)] (see Fig. 4). It is apparent that *sd*[*e*(*n*, *N*)] obtained on real data decreases faster than *sd*[*e*(*n*)] as *n* approaches to *N*, with increasing discrepancy, up to the point when it becomes zero for $n=N$, $sd[e(n=N,N)]=0$, whereas the true standard deviation estimation error for $n=N$ is strictly greater than zero $sd[e(n=N)]> 0$. In Fig. 4 it can also be seen that $sd[e(n)]\simeq sd[e(n,N)]$ for $n<<N$. This discrepancy observed for *n* approaching *N* will be referred to as “tail-effect”.

Finally, we will consider the systematic discrepancy from the true error caused by the tail effect and propose a fraction $n/N<20\%$ under which this approximation has a small impact on the results.

#### Quantifying the “tail-effect”

In the same mathematical framework used to develop Eq. (5), the “tail-effect” is quantified by following result.

##### Theorem 2

Under the same assumption of Theorem 1, for any *N* and *n*, $n=1,2,\ldots ,N$ it holds that:

6 $$\begin{aligned} \text {sd}[t(n)-t(N)] = \sqrt{\text {var}[t(N)] + \frac{N-2n}{N} \text {var}[t(n)] - 2\sigma ^2 \bigg ( \frac{\alpha }{nN} \frac{(1-\alpha ^{n})(1-\alpha ^{N-n}}{(1-\alpha )^2} \bigg )}. \end{aligned}$$

The proof is reported in the “Methods”.

Figure 4 illustrates the “tail effect”, by comparing *sd*[*e*(*n*)] and *sd*[*e*(*n*; *N*)] provided by Theorems 1 and 2, respectively. Different values of *N* are considered. Population values $\alpha ^*$ and $p_h^*$ are used. *sd*[*e*(*n*)], reported in dashed blue line, is clearly not dependent from *N*, and goes to zero for $n \rightarrow \infty$. *sd*[*e*(*n*; *N*)] provides different curves based on *N*: 1 month (yellow curve with diamonds), 4 months (red curve with triangles), 8 months (green curve with circles), 12 months (black curve with squares). It is well visible how the curves exhibit the “tail effect”: they decrease faster than *sd*[*e*(*n*)], thus leading to a systematic underestimation of the error. The systematic discrepancy is the larger the closer *n* approaches *N* until, the curve of *sd*[*e*(*n*; *N*)] becomes zero for $n=N$.

#### Determining an acceptable range of $n<<N$

**Figure 5:**
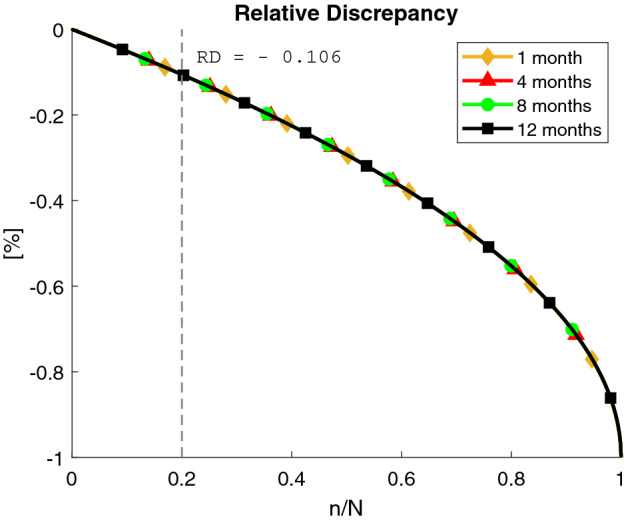
Relative Discrepancy between the standard deviation of the estimation error *sd*[*e*(*n*)] and its approximation *sd*[*e*(*n*, *N*)] affected by the tail effect. The curves are obtained for different trial durations: $N=1$ month (diamond yellow), $N=4$ months (triangle red), $N=8$ months (circle green), $N=12$ months (square black). Moreover, $\alpha =\alpha *=0.917$ and $p_h=p_h*=0.043$ are used.

We analyzed the systematic underestimation of *sd*[*e*(*n*)] introduced by the approximation ${{\hat{p}}}_h=t(N)$ in *sd*[*e*(*n*; *N*)] by considering the Relative Discrepancy (RD)

$$\begin{aligned} \text {RD}=\frac{sd[e(n;N)]-sd[e(n)]}{sd[e(n)]} \end{aligned}$$

that is, the relative magnitude of the discrepancy between the sd[e(n;N)] and the true error sd[e(n)], with respect to the true error.

In Fig. 5, RD is reported with respect to the trial fraction *n*/*N* for different *N* values: 1 month (yellow curve with diamonds), 4 months (red curve with triangles), 8 months (green curve with circles), 12 months (black curve with squares). Note that the discrepancy is always negative, confirming a systematic underestimation of the magnitude of the estimation error. RD curves obtained for different values of *N* overlap well, suggesting that the relative discrepancy does not depend on *N* but only on the fraction *n*/*N*.

As a result, it is possible to determine a fraction of *n*/*N* for which the relative discrepancy is negligible. In particular, for $n/N\le 0.2$ then $RD\le 0.11$. Thus if a systematic underestimation of at most $11\%$ is considered acceptable, and one is interested in evaluating on a new dataset the estimation error by computing the sample variance *SD*[*e*(*n*; *N*)], than *n* should not be larger than 20% of the total trial length *N*. For example, we have considered $n_{\max }=30$ days over $N=6$ months, i.e., $n \simeq 0.16 N$, which corresponds to a relative discrepancy of $RD=8.35\%$. According to Theorem 1, $sd[e(n)]=1.0\%$ for $n=30$ days (see Table 1). This means that using the formula of Theorem 2 we are underestimating the actual *sd*[*e*(*n*)] of $0.083\%$.

## Discussion

In diabetes management, CGM sensors are used by patients to continuously monitor BG in order to keep it inside the physiological range of (70–180) mg/dL. The fraction of time spent within or outside this range, namely the TBR considered in this paper but also the time in range (TIR) and the time above range (TAR), are indicators commonly adopted by clinicians and patients to assess therapy effectiveness. However, clinical reliability of these metrics requires sufficiently long CGM recordings.

In this work, we proposed a mathematical approach to determine the minimum CGM monitoring window which warrants a desired level of accuracy for TBR. Specifically, we derived a theorem that, under the assumption of autoregressive structure of order 1 for the dichotomized CGM data, provides a formula which links the accuracy of TBR estimation to the number of CGM samples in the recording. We considered outpatient CGM data collected in 148 adults with type 1 diabetes and successfully assessed the formula using either subject-specific parameters or population parameters.

The proposed formula of Eq. (5) links the number of CGM samples *n* to the uncertainty of time in hypoglycemia estimation, expressed as standard deviation of the estimation error $\text {sd}[e(n)]$. Thus, the formula can be used to compute $\text {sd}[e(n)]$ when examining data of a clinical trial of a certain duration *n*, providing a measure of reliability of the experimental findings (application 1). On the other hand, when designing a clinical trial, the formula can be used to determine a sufficient *n* granting to achieve a desirable accuracy $\text {sd}[e(n)]$ (application 2). Table 1 reports several couples of trial duration (expressed in number of days in the first row and CGM samples in the second row) and uncertainty (third row), obtained with the population $p_h^*$ and $\alpha ^*$ previously derived.

Examples of possible applications are reported below:

- *Application 1* When estimating the time in hypoglycemia spent by a subject monitored for *m* days, our formula allows evaluating the uncertainty of the estimate. For example, if a patient is monitored for *m* = 14 days (corresponding to *n* = 4032 samples for a CGM sensor providing 1 sample every 5 min) and shows $5\%$ of time in hypoglycemia, our formula suggests that 2-week monitoring grants the uncertainty of: $5\% \pm 1.5\%.$
- *Application 2* When designing a clinical trial, our formula can provide the minimum number of CGM samples *n* (i.e., the minimum trial duration) needed to reach a desirable estimation error. For example, if $1\%$ estimation error is deemed clinically acceptable, the proposed formula suggests to collect, at least, $n=8808$ CGM samples that, for a CGM sensor providing 1 sample every 5 min, equal to $m=30.6$ days.

**Table 1:** Trial duration in days (*m*, first row) and CGM samples (*n*, second row), and associated standard deviation of time in hypoglycemia estimation error (*sd*[*e*(*n*)], third row) obtained for the population under analysis ($p_h=0.043$ and $\alpha =0.917$), obtained using the proposed formula.

m (days)	7	14	30	60	120
n (CGM samples)	2016	4032	8640	17280	34560
sd (e(n)) (%)	2.1	1.5	1.0	0.7	0.5

In conclusion, the proposed formula can be used to determine the uncertainty of TBR estimated in a trial of a given duration and to determine the minimum duration of a clinical trial granting to achieve a desirable uncertainty in TBR.

**Figure 6:**
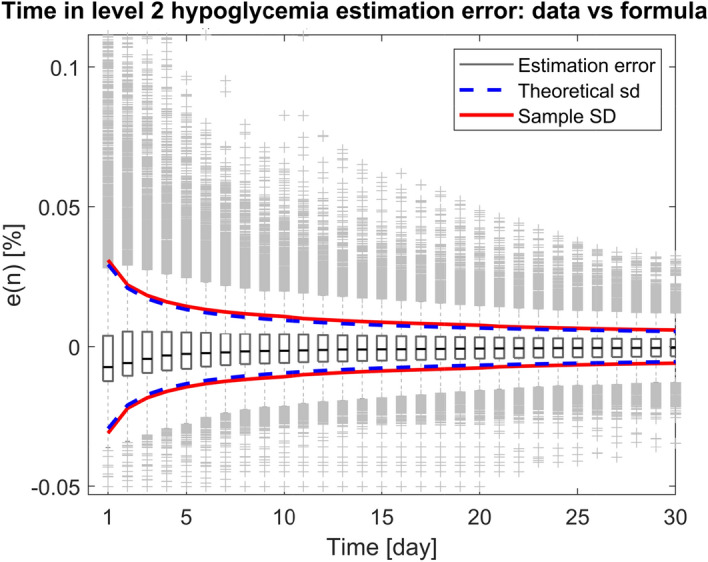
Estimation error of time in level 2 hypoglycemia, for different window durations, for the whole population under analysis. The standard deviation of the estimation error is computed both on CGM data (red) and by using the proposed formula of Eq. (5) (dashed blue).

It should be remarked that the proposed approach can be straightforwardly extended to the other TIRs metrics, thus strengthening its utility in the design of all those clinical trials where the duration of BG monitoring is particularly significant in clinical relevance as well as in cost-effectiveness terms.

As an example, this is illustrated for the so called “level 2 hypoglycemia” (L2H), defined as the percent time spent with $CGM<54$ mg/dL^14^. This metric focuses on glucose concentration levels even lower than the ones considered in TBR and has a demonstrated association to cognitive dysfunction, impaired awareness of hypoglycemia, cardiac arrhythmia and increased mortality^23^. To consider this new metric, we estimated ${{\hat{p}}}_h$ and ${\hat{\alpha }}$ as described above, and plug the newly obtained parameters into Eq. (5). Figure 6 reports the standard deviation of L2H estimation error predicted by the so-derived formula (dashed blue), and the sample standard deviation computed on the data (red). The two curves overlap well, showing that the methodology of this work results effective also for L2H.

As a final comment, note that the REPLACE-BG dataset was used here to illustrate the meaningfulness of the assumptions underlying our theorem. This dataset has a reasonable sample size and uses CGM sensors with reasonable accuracy but it is should be acknowledged that it includes only subjects treated with insulin pumps, without significant hypoglycemia unawareness and with low risk for the developing severe hypoglycemia. Therefore, future works will focus on the analysis of larger datasets, more representative of the overall T1D population, including a larger representation of hypoglycemia unawareness, subjects under different insulin regimens and subjects with larger incidence of hypoglycemia. Furthermore, the proposed formula will be also tested in other populations of people with diabetes (e.g., type 2 diabetes, pregnancy) and in specific sub-populations (e.g., young, adolescent).

## Methods

### Derivation of Theorem 1

#### Proof

Let us define ${\bar{h}}_i = h_i-p_h$ the zero-meaned version of $h_i$. Similarly, we can define ${\bar{t}}_n=\frac{1}{n} \sum _{i=1}^{n}{\bar{h}}_i$, so that the estimation error variance can be expressed as follows:

$$\begin{aligned} \begin{aligned} \text {var}[e(n)]&= \text {var}[{\bar{t}}(n)] \\&= {\mathbb {E}}\bigg [ \frac{1}{n} \sum _{k=1}^{n} {\bar{h}}_k \frac{1}{n} \sum _{\ell =1}^{n} {\bar{h}}_\ell \bigg ] \\&= \frac{1}{n^2} {\mathbb {E}} \bigg [ \sum _{k=1}^{n} {\bar{h}}_k^2 + 2 \sum _{\ell =1}^{n} \sum _{\ell>k}^{n} {\bar{h}}_k{\bar{h}}_\ell \bigg ] \\&= \frac{1}{n^2} \bigg ( n \sigma ^2 + 2 \sum _{k=1}^{n} \sum _{\ell >k}^{n} {\mathbb {E}}[{\bar{h}}_k{\bar{h}}_\ell ] \bigg ), \\ \end{aligned} \end{aligned}$$

where $\sigma ^2=p_h(1-p_h)$ is the variance of the Bernoulli variable ${\bar{h}}_i$.

The term ${\mathbb {E}}[{\bar{h}}_k{\bar{h}}_\ell ]$ represents the autocovariance function of $h_k$ that, under the hypothesis of AR(1) structure, can be rewritten as $\sigma ^2\alpha ^{\ell -k}$. So we obtain:

$$\begin{aligned} \text {var}[e(n)] = \frac{1}{n} \bigg ( \sigma ^2 + \frac{2}{n} \sigma ^2 \sum _{k=1}^{n} \sum _{\ell >k}^{n} \alpha ^{\ell -k} \bigg ). \end{aligned}$$

Substituting $\xi =\ell -k-1$ in the last summation:

$$\begin{aligned} \text {var}[e(n)] = \frac{\sigma ^2}{n} \bigg ( 1 + \frac{2}{n} \alpha \sum _{k=1}^{n} \sum _{\xi =0}^{n-k-1} \alpha ^\xi \bigg ). \end{aligned}$$

The second summation is the geometric sum of parameter $\alpha$, which converges to $\frac{1-\alpha ^{n-k}}{1-\alpha }$. Substituting $\nu =n-k$, we obtain:

$$\begin{aligned} \text {var}[e(n)] = \frac{\sigma ^2}{n} \bigg (1 + \frac{2 \alpha n}{n (1-\alpha )} - \frac{2\alpha }{n(1-\alpha )} \sum _{\nu =0}^{n-1} \alpha ^\nu \bigg ). \end{aligned}$$

Again, the second summation is the geometric sum of parameter $\alpha$. So we have:

$$\begin{aligned} \text {var}[e(n)] = \frac{\sigma ^2}{n} \bigg (1 + \frac{2 \alpha }{1-\alpha } + \frac{2\alpha }{n} \frac{\alpha ^n-1}{(1-\alpha )^2} \bigg ). \end{aligned}$$

$$\square$$

### Illustration of the results on synthetic data

To illustrate the correctness of the proposed formula, we generated synthetic data matching the assumptions driving our theorem.

**Figure 7:**
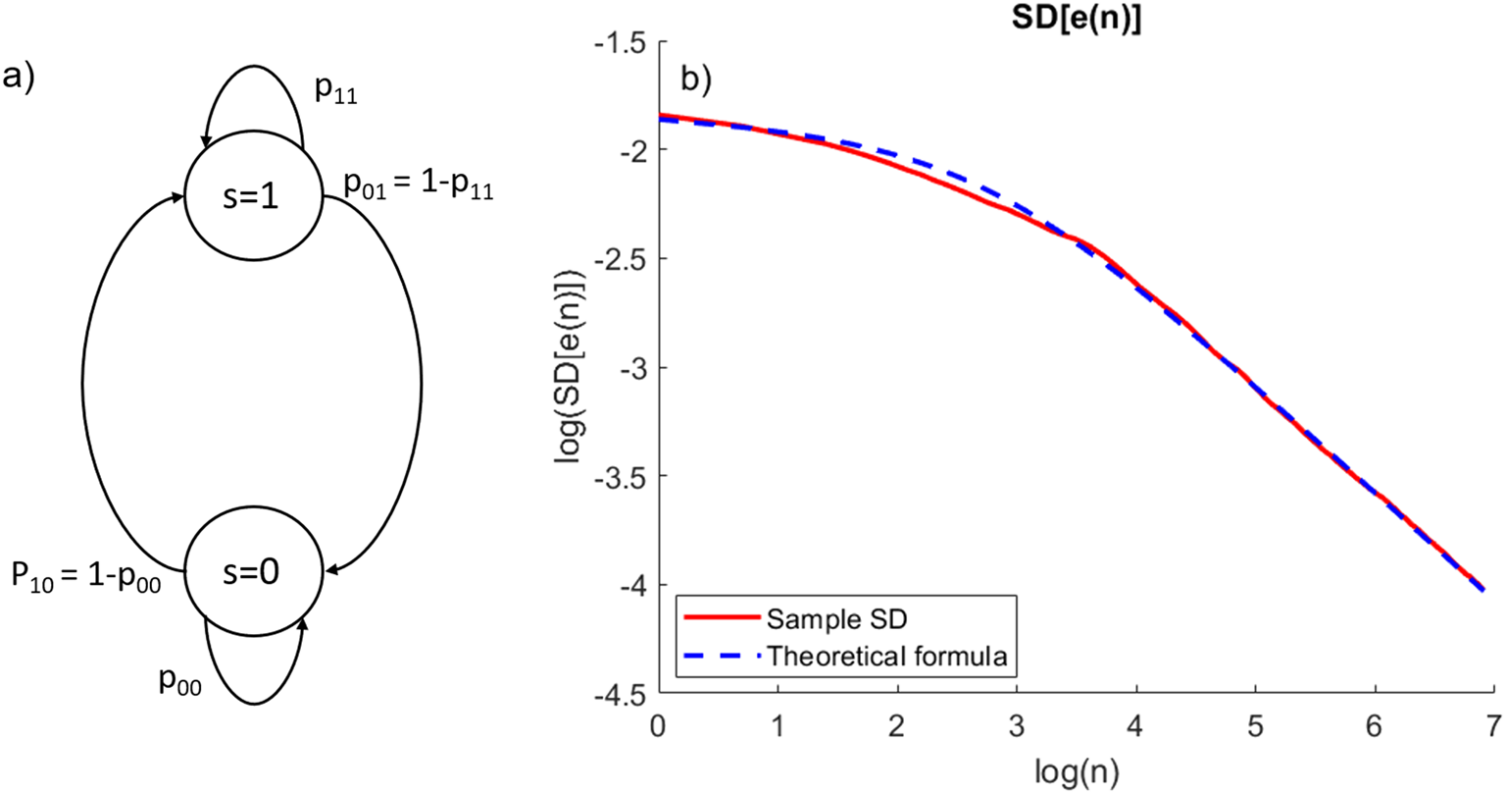
Synthetic data analysis. (**a**) Scheme of the Markov chain used to generate synthetic Bernoulli processes matching the assumptions driving our theorem. (**b**) Sample standard deviation of the estimation error *e*(*n*; *i*) computed over $N_{\text {rep}}=5000$ Bernoulli processes, each of $N=1000$ samples with $\alpha =0.86$ and $\sigma ^2=0.024$ (red). The sample standard deviation is predicted well by the proposed formula of Eq. (5) (dashed blue).

To generate a Bernoulli random process with user-defined parameters ($p_h, \alpha$), we implemented a time-homogeneous Markov chain with a finite state space, whose scheme is reported in Panel a of Fig. 7. The chain has two states: $s=1$ represents a sample in hypoglycemia, while $s=0$ reflects a non-hypoglycemic state. The probabilities of being in a state $s \in [0,1]$ ($p_0$, $p_1$) at time *k* evolve according to the following equations:

7 $$\begin{aligned} {\left\{ \begin{array}{ll} p_0(k) = p_{00} p_0(k-1) + (1-p_{11}) p_1(k-1) \\ p_1(k) = (1-p_{00}) p_0(k-1) + p_{11} p_1(k-1), \end{array}\right. } \end{aligned}$$

where $p_{ij}$ represents the transition probability of being at state $s=j$ at time $k+1$ starting from the state $s=i$ at time *k*. Since there are only two possible states, $p_{01}=1-p_{00}$ and $p_{10}=1-p_{11}$.

From Eq. (7) it is possible to determine the stationary probability of the chain $p({\infty })=[p_0(\infty ), p_1(\infty )]$, as a function of $p_{00}, p_{11}$. In particular, $p_1(\infty )$ represents the probability of hypoglycemia $p_h$. So, there is a unique link between the transition probabilities of the Markov chain $p_{00}, p_{11}$ and the probability of hypoglycemia $p_h$. Similarly, the transition probabilities can also be uniquely linked to the correlation parameter $\alpha$:

$$\begin{aligned} {\left\{ \begin{array}{ll} p_h = \frac{p_{00}-1}{p_{00}+p_{11}-2} \\ \alpha = \frac{p_{11}(p_{00}+p_{11}-2)}{p_{11}-1}-\frac{p_{00}-1}{p_{11}-1}. \end{array}\right. } \end{aligned}$$

The derivation of these relationships is provided in the Supplementary methods. By appropriately setting the values of $p_{00}, p_{11}$, it is therefore possible to control the values of $p_h,\alpha$, that are thus exactly known. In particular, the higher $p_{00}$ and $p_{11}$ absolute values, the more correlated the samples (e.g., $p_{00}=0.5$, $p_{11}=0.5$ provide the minimum correlation $\alpha =0$). Furthermore, the higher the difference between $p_{00}$ and $p_{11}$, the closer $p_h$ to 0 or 1 (e.g., $p_{00}=1$, $p_{11}=0$ provide $p_h=0$, while $p_{00}=0$, $p_{11}=1$ provide $p_h=1$).

We then used the above described Markov chain to generate a random process $h_k$ of *N* correlated Bernoulli variables, simulating the dichotomized glucose measurements of a subject. On this trace, we computed the estimate *t*(*n*), i.e., the sample mean over the first *n* samples, and the estimation error $e(n)=t(n)-p_h$. This is done for various lengths of the averaging window $n = 1,\ldots ,N$.

We replicated this procedure $N_{\text {rep}}$ times to simulate a clinical trial of $N_{rep}$ subjects, thus generating $N_\text {rep}$ realizations of the random process $h_k$.

Following this procedure, for each value of the window length $n=1,\ldots , N$ we have $N_{\text {rep}}$ realization of the estimation error *e*(*n*):

$$\begin{aligned} e(n;i)=t(n;i)-p_h, \qquad i=1, \ldots ,N_{\text {rep}} \end{aligned}$$

and we can evaluate the standard deviation of e(n), $\text {sd}[e(n)]$, by considering the sample standard deviation *SD* over the $N_{\text {rep}}$ realizations:

$$\begin{aligned} \text {SD}[e(n)]=\sqrt{\frac{1}{N_{\text {rep}}-1}\sum ^{N_{\text {rep}}}_{i=1}e(n;i)^2}. \end{aligned}$$

The approach is described in detail in the pseudo-code reported below.

Figure 7 shows the sample standard deviation (solid red line) obtained after the generation of $N_\text {rep}=5000$ Bernoulli processes of $N=1000$ samples each, with $\alpha =0.86$ and $\sigma ^2$ = 0.024. The curve matches well the standard deviation obtained with the proposed formula, illustrating the correctness of our finding. A log-log scale is used to better represent both the quantities.
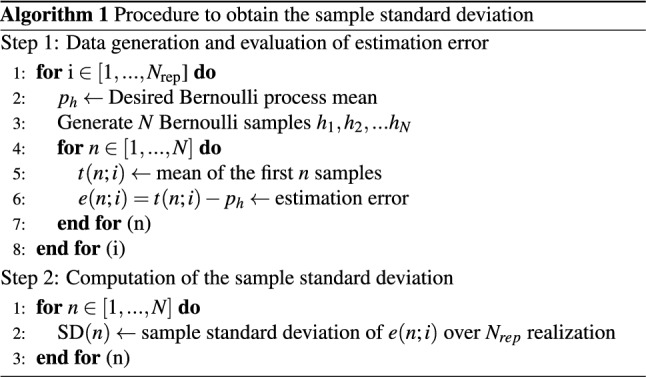

### Derivation of Theorem 2

#### Proof

8 $$\begin{aligned} \begin{aligned} \text {var}[t(n)-t(N)]&= {\mathbb {E}} \bigg [ \bigg ( \frac{1}{n} \sum _{k=1}^{n}{\bar{h}}_k \bigg ) - \bigg ( \frac{1}{N} \sum _{\ell =1}^{N}{\bar{h}}_\ell \bigg ) \bigg ]^2 \\&= \underbrace{{\mathbb {E}}\bigg [\frac{1}{n} \sum _{k=1}^{n} {\bar{h}}_k\bigg ]^2}_{\text {var}\big [t(n)\big ]} + \underbrace{{\mathbb {E}}\bigg [\frac{1}{N}\sum _{\ell =1}^{N} {\bar{h}}_\ell \bigg ]^2}_{\text {var}\big [t(N)\big ]} - 2 \underbrace{ {\mathbb {E}}\bigg [\frac{1}{n}\sum _{k=1}^{n}{\bar{h}}_k\frac{1}{N}\sum _{\ell =1}^{N}{\bar{h}}_\ell \bigg ]}_{\text {cov}\big [t(n),t(N)\big ]}. \end{aligned} \end{aligned}$$

The first two terms of the sum can be obtained by Eq. (5). The last term of the sum represents the covariance of *t*(*n*) and *t*(*N*), that can be rewritten as follows:

9 $$\begin{aligned} \begin{aligned} \text {cov}[t(n),t(N)]&= {\mathbb {E}}\bigg [ \frac{1}{nN} \bigg ( \sum _{k=1}^{n} {\bar{h}}_k \bigg ) \bigg ( \sum _{\ell =1}^{N} {\bar{h}}_\ell \bigg ) \bigg ] \\&= {\mathbb {E}}\bigg [ \frac{1}{nN} \bigg ( \sum _{k=1}^{n} {\bar{h}}_k \bigg ) \bigg ( \sum _{\ell =1}^{n} {\bar{h}}_\ell + \sum _{\ell =n+1}^{N} {\bar{h}}_\ell \bigg ) \bigg ] \\&= \frac{1}{nN} {\mathbb {E}} \bigg [ \bigg ( \sum _{k=1}^{n} {\bar{h}}_k \bigg )^2 + \bigg ( \sum _{k=1}^{n} {\bar{h}}_k \bigg ) \bigg ( \sum _{\ell =n+1}^{N} {\bar{h}}_\ell \bigg ) \bigg ] \\&= \frac{n}{N} \text {var}[t(n)] + \frac{1}{nN} {\mathbb {E}} \bigg [ \sum _{k=1}^{n} \sum _{\ell =n+1}^{N} {\bar{h}}_k{\bar{h}}\ell \bigg ] \\&= \frac{n}{N} \text {var}[t(n)] + \frac{\sigma ^2}{nN} \sum _{k=1}^{n} \sum _{\ell =n+1}^{N} \alpha ^{\ell -k}, \\&= \frac{n}{N} \text {var}[t(n)] + \frac{\sigma ^2}{nN} \sum _{k=1}^{n} \alpha ^{-k} \sum _{\ell =n+1}^{N} \alpha ^{\ell } \\&= \frac{n}{N} \text {var}[t(n)] + \frac{\sigma ^2}{nN} \sum _{k=1}^{n} \alpha ^{-k} \sum _{\xi =0}^{N-n-1} \alpha ^{\xi +n+1}, \quad \xi =\ell -n-1 \\&= \frac{n}{N} \text {var}[t(n)] + \frac{\sigma ^2}{nN} \sum _{k=1}^{n} \alpha ^{-k+n+1} \frac{1-\alpha ^{N-n}}{1-\alpha } \\&= \frac{n}{N} \text {var}[t(n)] + \frac{\sigma ^2 \alpha (1-\alpha ^{N-n})}{nN (1-\alpha )} \sum _{k=1}^{n} \alpha ^{n-k} \\&= \frac{n}{N} \text {var}[t(n)] + \frac{\sigma ^2 \alpha (1-\alpha ^{N-n})}{nN (1-\alpha )} \sum _{\nu =0}^{n-1} \alpha ^{\nu }, \quad \nu =n-k \\&= \frac{n}{N}\text {var}[t(n)]+\frac{\sigma ^2 \alpha }{nN} \frac{(1-\alpha ^n)(1-\alpha ^{N-n})}{(1-\alpha )^2}. \end{aligned} \end{aligned}$$

By joining Eqs. (8) and (9) we obtain the expression of Theorem 2. $\square$

This formula was verified both in a controlled environment (using a Markov-chain model to build synthetic Bernoulli processes) and over real CGM data. In both cases, it was proven to accurately model the estimation error $e(n;N), n=1,2,\ldots ,N$.

## Supplementary information

Supplementary Information.

## Data Availability

Data can be obtained from the T1D Exchange archive (https://t1dexchange.org/research/biobank/). All the scripts for implementing the methodology in Matlab are publicly available at https://github.com/NunzioCamer/AnalyticalTBRestimation.
