# Supplementary material for: An analytical approach to determine the optimal duration of continuous glucose monitoring data required to reliably estimate time in hypoglycemia
Source: Sci Rep. 2020 Oct 23;10:18180. doi: 10.1038/s41598-020-75079-5 (PMC7584616; doi:10.1038/s41598-020-75079-5)
Supplement: Supplementary file 1 — Supplementary Information. [file 41598_2020_75079_MOESM1_ESM.pdf]

An analytical approach to determine the optimal duration of continuous glucose monitoring data required to reliably estimate time in hypoglycemia

Supplementary Information

Nunzio Camerlingo[1], Martina Vettoretti[1], Andrea Facchinetti[1], Giovanni Sparacino[1], Julia K. Mader[2], Pratik Choudhary[1], and Simone Del Favero[1*]

[1]University of Padova, Department of Information Engineering, Padova, 35131, Italy
[2]Medical University of Graz, Division of Endocrinology and Diabetology, Graz, 8036, Austria
[3]King's College London, Department of Diabetes, School of Life Course Sciences, London, SE59RJ, United Kingdom

[*]sdelfave@dei.unipd.it

Supplementary methods

Implementation of a Markov chain to generate Bernoulli processes

To generate synthetic data matching the assumptions driving our theorem, we implemented a time-homogeneous Markov chain with a finite state space. The chain has two possible states $s \in [0,1]$, mimicking the samples of the dichotomized CGM trace h_k. The probabilities of being in a state s at time k are described by:

$$\mathbf{p}(k) = T(k \mid k-1)\mathbf{p}(k-1), \qquad T(k \mid k-1) = \begin{bmatrix} p_{00} & 1-p_{11} \\ 1-p_{00} & p_{11} \end{bmatrix}$$

where p_{ij} represents the transition probability of being at state $s = j$ at time $k+1$ starting from the state $s = i$ at time k, and $T(k \mid k-1)$ is known as left stochastic matrix.

In a time-homogeneous Markov chain, T is time-invariant, i.e., $T(k \mid k-1) = T$, $\forall k = 1,2,\ldots,\infty$, therefore:

$$p(\infty) = Tp(\infty) \qquad \rightarrow \qquad (T - I_2)p(\infty) = 0 \tag{1}$$

where I_2 is the identity matrix of dimension 2×2 and $p(\infty) = [p_0(\infty), p_1(\infty)]^T$ is known as stationary distribution of the chain (note that $p(\infty)$ is an eigenvector of $T - I$).
From Eq. (1) the following relationship can be written:

$$(1 - p_{00})p_0(\infty) + (p_{11} - 1)p_1(\infty) = 0$$

Since $p_0(\infty) = 1 - p_1(\infty)$, we have:

$$p_1(\infty) = \frac{p_{00} - 1}{p_{00} + p_{11} - 2} \tag{2}$$

that, in our framework, represents the parameter p_h.

The covariance of two following states s_k, s_{k+1}, $k = 1,2,...,\infty$ is given by:

$$\begin{aligned}
\mathrm{cov}[s_k, s_{k+1}] &= \mathbb{E}[s_k, s_{k+1}] - \mathbb{E}^2[s_k] \\
&= \sum_{i=0}^{1}\sum_{j=0}^{1} ijp(s_k = i, s_{k+1} = j) - p(s_k = i)p(s_k = j) \\
&= p(s_k = 1, s_{k+1} = 1) - p^2(s_k = 1)
\end{aligned}$$

By definition of conditional probability we obtain:

$$\begin{aligned}\mathrm{cov}[s_k, s_{k+1}] &= p(s_{k+1}=1|s_k=1)p(s_k=1) - p^2(s_k=1)\\ &= p_{11}p_1(k) - p_1^2(k)\end{aligned}$$

Substituting Eq. (2) in $p_1(k)$:

$$\mathrm{cov}[s_k, s_{k+1}] = p_{11}\frac{p_{00}-1}{p_{00}+p_{11}-2} - \left(\frac{p_{00}-1}{p_{00}+p_{11}-2}\right)^2$$

To force an AR(1) structure for s_k, the following expression must be verified:

$$\mathrm{cov}[s_k, s_{k+1}] = p_h(1-p_h)\alpha$$

Substituting Eq. (2) in p_h we obtain:

$$\alpha = \frac{p_{11}(p_{00}+p_{11}-2)}{p_{11}-1} - \frac{p_{00}-1}{p_{11}-1} \tag{3}$$

Eqs. (2-3) represent a unique link between the transition probabilities p_{00}, p_{11} and the parameters p_h, α. Thus, p_h, α can be controlled and exactly known by setting the values of p_{00}, p_{11}.

Supplementary notes

Details of REPLACE-BG dataset

People with diabetes need to closely monitor glucose concentration, in order to guide their therapy and keep their blood glucose (BG) in the recommended target range[1]. Fingerstick BG testing has been the most commonly used method of self-monitoring of BG (SMBG) in diabetes management[2]. Subjects under SMBG therapy are recommended to collect episodic BG readings: at least 2 times per day, or more frequently, based on diabetes type and glycemic control[3]. Despite this strategy provides a very accurate BG value, frequent BG testing is often cumbersome for patients.

Continuous glucose monitoring (CGM) technology represents an alternative for monitoring glucose concentration, which prevents the need for frequent pricks. CGM uses electrochemical sensors that enable continuous interstitial fluid glucose monitoring. Therefore, it can provide the glucose trend in real time and alert the user of hyper- and hypoglycemia[4].

Since the first CGM were not as accurate as fingerprick, some clinicians recommended patients to use CGM in adjuncte to SMBG, i.e., before making the treatment decisions, they should check the CGM reading by confirmatory fingerstick. In recent years, the technological developments and the enhancement of signal processing algorithms led the CGM sensors reaching the range of accuracy performance of SMBG device[5], and the consequent regulatory approval of CGM nonadjunctive use, i.e., the use of CGM readings to make treatment decisions without confirmatory fingersticks. Nevertheless, some clinicians still recommend its adjunctive use.

The REPLACE-BG trial[6] was conduced to determine whether the routine use of CGM without fingerstick confirmation was as safe and effective as CGM used as an adjunct to the BG measurement. The study involved 225 adults with type 1 diabetes, randomly assigned either to the nonadjunctive group ($N = 148$) or the adjunctive one ($N = 77$), and monitored up to 6 months. For our analysis, we needed to consider only a single treatment, in order to obtain parameters (α^*, p_h^*) as much representative as possible of the population under analysis. Specifically, we selected the nonadjunctive arm, since it is the most numerous one, but the same analysis can be repeated, separately, for the other arm.

Participants under the nonadjunctive group were 71 women, and 44 ± 14 years old, with mean diabetes duration of 23 ± 12 months. They wore a Dexcom G4 Platinum sensor (Dexcom, Inc), measuring the interstitial glucose every 5 min for up to 7 days with an enhanced algorithm (Software 505) which improved the sensor accuracy[7].

The REPLACE-BG dataset can be downloaded on[8].

References

1. Cappon, G., Vettoretti, M., Sparacino, G. & Facchinetti, A. Continuous glucose monitoring sensors for diabetes management: a review of technologies and applications. *Diabetes & Metab. J.* **43**, 383–397 (2019).

2. Clarke, S. & Foster, J. A history of blood glucose meters and their role in self-monitoring of diabetes mellitus. *Br. J. Biomed. Sci.* **69**, 83–93 (2012).

3. Bailey, T. S. *et al.* American association of clinical endocrinologists and american college of endocrinology 2016 outpatient glucose monitoring consensus statement. *Endocr. Pract.* **22**, 231–261 (2016).

4. Mian, Z., Hermayer, K. L. & Jenkins, A. Continuous glucose monitoring: review of an innovation in diabetes management. *The Am. J. Med. Sci.* (2019).

5. Facchinetti, A. Continuous glucose monitoring sensors: past, present and future algorithmic challenges. *Sensors* **16**, 2093 (2016).

6. Aleppo, G. *et al.* Replace-bg: a randomized trial comparing continuous glucose monitoring with and without routine blood glucose monitoring in adults with well-controlled type 1 diabetes. *Diabetes Care* **40**, 538–545 (2017).

7. Bailey, T. S., Chang, A. & Christiansen, M. Clinical accuracy of a continuous glucose monitoring system with an advanced algorithm. *J. Diabetes Sci. Technol.* **9**, 209–214 (2014).

8. T1d exchange biobank. https://t1dexchange.org/research/biobank/. Accessed: 2019-12-30.
